# Supervised, Multivariate, Whole-Brain Reduction Did Not Help to Achieve High Classification Performance in Schizophrenia Research

**DOI:** 10.3389/fnins.2016.00392

**Published:** 2016-08-25

**Authors:** Eva Janousova, Giovanni Montana, Tomas Kasparek, Daniel Schwarz

**Affiliations:** ^1^Institute of Biostatistics and Analyses, Faculty of Medicine, Masaryk UniversityBrno, Czech Republic; ^2^Department of Biomedical Engineering, King's College LondonLondon, UK; ^3^Behavioural and Social Neuroscience Group, CEITEC - Central European Institute of Technology, Masaryk UniversityBrno, Czech Republic; ^4^Department of Psychiatry, University Hospital Brno and Masaryk UniversityBrno, Czech Republic

**Keywords:** computational neuroanatomy, pattern recognition, classification, penalized linear discriminant analysis, support vector machines, cross-validation, magnetic resonance imaging, schizophrenia

## Abstract

We examined how penalized linear discriminant analysis with resampling, which is a supervised, multivariate, whole-brain reduction technique, can help schizophrenia diagnostics and research. In an experiment with magnetic resonance brain images of 52 first-episode schizophrenia patients and 52 healthy controls, this method allowed us to select brain areas relevant to schizophrenia, such as the left prefrontal cortex, the anterior cingulum, the right anterior insula, the thalamus, and the hippocampus. Nevertheless, the classification performance based on such reduced data was not significantly better than the classification of data reduced by mass univariate selection using a *t*-test or unsupervised multivariate reduction using principal component analysis. Moreover, we found no important influence of the type of imaging features, namely local deformations or gray matter volumes, and the classification method, specifically linear discriminant analysis or linear support vector machines, on the classification results. However, we ascertained significant effect of a cross-validation setting on classification performance as classification results were overestimated even though the resampling was performed during the selection of brain imaging features. Therefore, it is critically important to perform cross-validation in all steps of the analysis (not only during classification) in case there is no external validation set to avoid optimistically biasing the results of classification studies.

## Introduction

Schizophrenia is a disabling chronic psychiatric disease characterized by symptoms such as hallucinations, delusions, thought disturbances, or poverty of speech (Liddle, 1987). It affects young people in their productive years (i.e., typically in their late teens or early twenties; Andreasen, 1995); the lifetime prevalence of schizophrenia is ~0.48% (Simeone et al., 2015). Thus, schizophrenia is one of the leading contributors to the global disease burden. Despite decades of schizophrenia etiology and manifestation research, its diagnostics is still based on clinical interviews that are subjective and sometimes inaccurate. There are accordingly endeavors to employ computer-assisted methods based on brain imaging data for schizophrenia diagnostics in order to reduce subjectivity and also increase the speed of diagnostic assessments (Klöppel et al., 2012; Wolfers et al., 2015).

Modern neuroimaging techniques have enabled the identification of brain regions in which schizophrenia patients show significant group differences compared with healthy subjects (Wright et al., 2000; Shenton et al., 2001; Niznikiewicz et al., 2003; Honea et al., 2005; Haijma et al., 2013). On individual levels, however, brain-imaging measurements in schizophrenics exhibit a wide overlap with the normal range (Sun et al., 2009), which makes the diagnosis of schizophrenia based on individual brain-image data quite demanding. The first studies that sought to distinguish schizophrenia patients from healthy controls (HC) based on magnetic resonance imaging (MRI) data were based on brain anatomical measures of a few selected regions of interest (Leonard et al., 1999; Nakamura et al., 2004). Later studies attempted to use whole-brain data for schizophrenia classification since the neural mechanisms of schizophrenia are not restricted to a certain region of the brain but are instead distributed (Michael et al., 2008; Melonakos et al., 2011). For example, some studies utilized voxel-based morphometry or other univariate whole-brain methods to select imaging features discriminating strongly between schizophrenia subjects and HC and use them for classification (Kovalev et al., 2003; Diaz et al., 2010). Such feature selection makes it possible to increase the signal-to-noise ratio and also avoid the so-called “small sample size problem” (Lemm et al., 2011) when the number of subjects is considerably smaller than the number of features, which often causes unstable classification performance (Demirci et al., 2008). Selecting imaging features based on feature-by-feature tests does not allow utilizing information about feature correlation, however. As a result, multivariate, whole-brain data reduction techniques are preferred. The most commonly used multivariate method in schizophrenia research is principal component analysis (PCA; Yoon et al., 2007; Karageorgiou et al., 2011). Nevertheless, PCA also has a few disadvantages: It is sometimes ineffective at capturing complex relationships in high-dimensional spaces since it is a linear method (Fan et al., 2005), and it is not guaranteed that the selected principal components corresponding to the largest eigenvalues are those discriminating the best between patients and HC since PCA does not take in account information about the patient and control group labels (Bunea et al., 2011; Janousova et al., 2015). To overcome these shortcomings, recent studies have used deep learning methods (Plis et al., 2014), normative modeling (Marquand et al., 2016), or supervised, multivariate, whole-brain reduction techniques that utilize group labels during data reduction, such as sparse multinomial logistic regression, a ν-multiple kernel learning approach and sparse network-based models used for MRI or functional MRI classification in schizophrenia research (Sun et al., 2009; Castro et al., 2014; Rosa et al., 2015). In other fields of study, supervised, multivariate, whole-brain reduction methods such as penalized least squares regression or sparse logistic regression have been used to classify HIV-infected individuals and music vs. speech stimuli in healthy subjects, respectively (Ryali et al., 2010; Bunea et al., 2011). In our previous imaging-genetics study of patients with Alzheimer's disease, we successfully used penalized linear discriminant analysis (pLDA) with resampling to pre-select image phenotypes (Vounou et al., 2012).

Here, we use pLDA with resampling to select the most discriminative features from MRI data to differentiate first-episode schizophrenia (FES) patients from HC. We investigate if this supervised, multivariate, whole-brain reduction method is capable of efficient data reduction that results in significantly better classification performance than mass univariate selection of features using a *t*-test or unsupervised, multivariate reduction using PCA. Moreover, we also examine the influence of the type of features extracted from the MRI data, namely gray matter (GM) volumes and local deformations, and the type of classifier on the classification performance. To enable comparison with other studies, we use two of the most commonly applied classifiers in schizophrenia research: linear discriminant analysis (Leonard et al., 1999; Nakamura et al., 2004; Takayanagi et al., 2010; Karageorgiou et al., 2011; Ota et al., 2012) and linear support vector machines (SVM; Yoon et al., 2007; Pohl and Sabuncu, 2009; Mourao-Miranda et al., 2012; Nieuwenhuis et al., 2012; Dluhos et al., 2014). Furthermore, we explore the effect of leave-one-out cross-validation (LOOCV) performed during classification solely or during data reduction and classification. The algorithm employs resampling during data reduction and therefore the brain imaging features are not selected from all of the input imaging data but rather from a number of random data subsets. Such an approach might enable to avoid optimistically biased classification results that occur in cases in which cross-validation is executed only during data classification.

The remainder of the paper is organized as follows. In Section Materials and Methods, we describe a data set of FES patients and HC used in our study, the preprocessing of their MRI data leading to the extraction of two sets of imaging features, the reduction of the feature sets using pLDA with resampling, and the subsequent classification using two classifiers including cross-validation. Section Results is dedicated to a summary of our classification results. Section Discussion discusses our results, compares them with classification performance based on commonly used data reduction techniques, and concludes the paper.

## Materials and methods

### Subjects

Our patient group included 52 males (median age: 23 years, range: 17–40 years) hospitalized in the all-male unit of the Department of Psychiatry, Masaryk University in Brno, Czech Republic; this cohort constituted the FES group. The diagnosis was established during a clinical interview performed by a trained psychiatrist. The interview was conducted in compliance with the International Statistical Classification of Disease and Related Health Problems (ICD-10) research criteria and was focused on information about family and personal history, somatic conditions, pharmacological history and current treatment, substance abuse, previous psychiatric conditions, the current clinical manifestation, symptoms and their duration, and functional impact. Where possible, information from relatives was collected as well. After the interview, the patients were physically examined, inclusive of a urine analysis (biochemistry, toxicology) and laboratory blood tests (hematology, biochemistry). Abnormal findings were subjected to additional tests and examinations. A fully trained senior psychiatrist reviewed all information, established the diagnosis and suggested the case for inclusion in the study. The exclusion criteria included substance dependence (detected via urine toxicology tests and clinical evaluation), neurological, or systemic disease with known relationships to brain alteration (detected by clinical evaluation, neurological and physical examinations, serum and urine chemistry, and blood count, serological examination for neurotropic agents and clinical evaluation of MRI scans), and contraindications for MRI examination.

The control group consisted of 52 HC matched for age (median age: 23 years, range: 18–38 years) and sex (all males) who were recruited from the community, medical staff, and students. Exclusion criteria included substance dependence, a personal or family history of mental illness, somatic conditions affecting the function or structure of the brain as assessed during clinical interviews performed by a trained psychiatrist, and contraindications for MRI.

The study was approved by the local ethics committee, and all subjects signed an informed consent form prior to their participation in the study.

### MRI data acquisition and preprocessing

All 104 subjects were scanned with a 1.5T Siemens Symphony machine. We acquired whole-head T1-weighted images using three-dimensional acquisition with IR/GR sequence, TR 1700 ms, TE 3.93 ms, TI 1100 ms, flip angle 15°. The sagittal tomographic plane thickness was 1.17 mm, the in-plane resolution was 0.48 × 0.48 mm, the 3-D field of view contained 160 × 512 × 512 voxels. We performed the MRI examination during the first episode, which means that the duration of treatment with antipsychotics was only from 3 to 14 weeks at the time of the MRI.

Prior to the analysis, we checked all of the images for morphological abnormalities. Since no abnormalities were found, we preprocessed all 104 images using Statistical Parametric Mapping 8 (SPM8: http://www.fil.ion.ucl.ac.uk/spm/). Specifically, the images were corrected for bias-field inhomogeneity, spatially normalized (i.e., transformed into stereotactic space), resampled to 1.5 × 1.5 × 1.5 mm, and segmented into GM, white matter and cerebrospinal fluid. The spatially normalized images were oriented axially and consisted of 170 × 256 × 256 voxels at 1.5 mm isotropic resolution. We modulated the GM tissue segment with the determinant of Jacobian matrices of the deformations to account for registration-related changes in local volumes, and we smoothed the data using an 8-mm full width at half maximum Gaussian kernel according to the optimized voxel-based morphometry (oVBM) procedure (Good et al., 2001). The GM volumes that we obtained were then used as the first type of features in the analysis.

The second type of features included local deformations that revealed how the brain anatomy of a diagnosed subject differs from the normal template anatomy in terms of local volume expansions and contractions. These data were acquired using deformation-based morphometry (DBM). The spatial normalization steps for DBM included the same affine registration algorithm as for oVBM apart from the resolution (1 × 1 × 1 mm). After transforming all bias-corrected images into stereotactic space, we used our original high-dimensional nonlinear registration technique (Schwarz et al., 2007) to compute vector displacement fields that maximized the normalized mutual information between the images and the high-resolution digital brain atlas. The resulting three-dimensional displacement vector fields were converted into scalar fields (referred to as “local deformations”) by computing the logarithms of their respective determinants of Jacobian matrices at each voxel in stereotactic space.

The final preprocessing step removed features corresponding to non-brain tissues and image background from the two feature sets. Each feature set from every subject was then transformed into one-dimensional vectors and arranged in an (*n*×*p*) matrix **X**, where *n* is the number of individuals in a data set and *p* is the number of features. The feature matrix of the GM volumes had a size of (104 × 748, 931); the dimensions of the feature matrix of the local deformations were (104 × 1, 924, 670).

### Imaging feature selection using pLDA with resampling

Since the number of features in the data set was very high even after the removal of non-brain and background features, both feature sets were reduced prior to classification to avoid the so-called “small sample size problem,” to increase the signal-to-noise ratio and possibly prevent for unstable classification results with poor generalizability (Demirci et al., 2008; Lemm et al., 2011). We used pLDA with resampling to identify the most discriminative features. The pLDA is described in detail elsewhere (Vounou et al., 2012) and is briefly summarized below.

In pLDA, a lasso penalty is imposed on the *l*_1_ norm of the direction vector **v** (Witten and Tibshirani, 2011) that best discriminates two classes within a data sample by maximizing the between-class variance and simultaneously minimizing the within-class variance. The direction vector **v** is a solution of the following optimization problem in the case of the common Fisher's LDA:

(1) $$\max_{v}\left\{v^{T}S_{B}v\right\}\text{ subject to }v^{T}S_{W}v=1,$$

where **S**_*B*_ and **S**_*W*_ are the between-class scatter matrix and the within-class scatter matrix, respectively, which can be calculated as:

(2) $$\begin{array}{l} S_{B}={\left(m_{H}-m_{D}\right)}^{T}\left(m_{H}-m_{D}\right),\\ S_{W}=\sum_{i\;=\;1}^{n_{H}}{\left(x_{i.}-m_{H}\right)}^{T}\left(x_{i.}-m_{H}\right)\\ \;+\sum_{i\;=\;1}^{n_{D}}{\left(x_{i.}-m_{D}\right)}^{T}\left(x_{i.}-m_{D}\right), \end{array}$$

where $m_{H}=1/n_{H}\;\cdot \;\sum_{i\;=\;1}^{n_{H}}x_{i.}$ is the mean vector of class *H* (healthy controls), $m_{D}=1/n_{D}\;\cdot \;\sum_{i\;=\;1}^{n_{D}}x_{i.}$ is the mean vector of class *D* (diseased individuals), *n*_*H*_ and *n*_*D*_ are the numbers of subjects in classes *H* and *D*, respectively, and **x**_*i*._, *i* = 1, …, *n*, are the rows of the feature matrix **X**.

In pLDA, imposing the lasso penalty leads to setting the coefficients *v*_*j*_, *j* = 1, …, *p*, of the least discriminative features to zero. Therefore, the optimization problem, which is solved by the minorization-maximization algorithm, changes to:

(3) $$\max_{v}\;\{v^{T}S_{B}v-\lambda \sum_{j\;=\;1}^{p}s_{j}\left|v_{j}\right|\}\text{ subject to }v^{T}S_{W}^{*}v=1,$$

where $S_{W}^{*}$ is the diagonal estimate of **S**_*W*_, diag $\left(S_{W}^{*}\right)\;=\;\left(s_{1}^{2},\ldots ,s_{p}^{2}\right)$, to avoid problems with possible singularity of **S**_*W*_, and λ is a regularization parameter that controls the degree of sparsity in the model. Specifically, when λ is exactly zero, no penalty is imposed and all *p* features contribute in the direction vector **v**. As λ increases above zero, fewer features contribute in **v**. At its maximum value, all coefficients of **v** are set to zero.

For the particular data, the optimal value of λ is not known a priori. Tuning λ commonly involves cross-validating the prediction error for a grid of values of λ and selecting the value of λ that leads to the smallest cross-validated error. However, this approach may be prone to sampling errors. Therefore, we combined the pLDA with a resampling method proposed in Meinshausen and Bühlmann (2010) for sparse predictive modeling. This procedure aims to calculate the selection probabilities *P*_*j*_(λ) for each feature by repeatedly fitting the pLDA model on random subsets of the data set while keeping track of the features associated with the non-zero coefficients of **v**. Specifically, an indicator variable $c_{\;}^{\left(k\right)}\left(\lambda \right)=\;\left(c_{1}^{\left(k\right)}\left(\lambda \right),\ldots ,c_{p}^{\left(k\right)}\left(\lambda \right)\right)$ is calculated in each iteration *k* = 1, …, *K*, where $c_{j}^{\left(k\right)}\left(\lambda \right)$, *j* = 1, …, *p*, is equal to 1 if the *j*th feature is selected (i.e., its respective coefficient *v*_*j*_ is non-zero) and 0 otherwise. Using all *K* random subsets, the selection probability for each feature can then be calculated as:

(4) $$P_{j}\left(\lambda \right)=\frac{1}{K}\sum_{k\;=\;1}^{K}c_{j}^{\left(k\right)}\left(\lambda \right),j=1,\ldots ,p.$$

The final set of the most discriminative features consists of those features with selection probabilities above a predefined threshold, e.g., 0.99.

To compare the results with common data reduction techniques, we also performed feature extraction using PCA (all *n* − 1 principal components corresponding to non-zero eigenvalues were used for classification) and feature selection using the Student's *t*-test with three different thresholds (*p* < 0.01, *p* < 0.005, and *p* < 0.001).

### Imaging feature classification

The final set of features selected using pLDA with resampling was then used for classifying individuals into the *D* or *H* class using two most commonly employed classifiers (i.e., LDA and linear SVM).

Since there was no external testing data set available, we used the LOOCV procedure to avoid overestimating the classification results. This procedure contains a loop in which one subject is chosen as a testing one and the remaining *n* − 1 subjects are employed for training the classifier. The testing subject is then classified as belonging to the patient or HC class, and the resulting class label is compared with the true classification label. This procedure is repeated *n* times using all subjects as the testing subject sequentially. Afterwards, the overall classification performance measures of accuracy, sensitivity, and specificity are calculated, which are defined as:

(5) $$\text{accuracy}=\frac{TP+TN}{TP+TN+FP+FN},$$

(6) $$\text{sensitivity}=\frac{TP}{TP+FN},$$

(7) $$\text{specificity}=\frac{TN}{TN+FP},$$

where *TP* and *TN* refer to numbers of true positive and true negative results, respectively, and *FP* and *FN* denote the numbers of false positive and false negative results, respectively.

**Figure 1:**
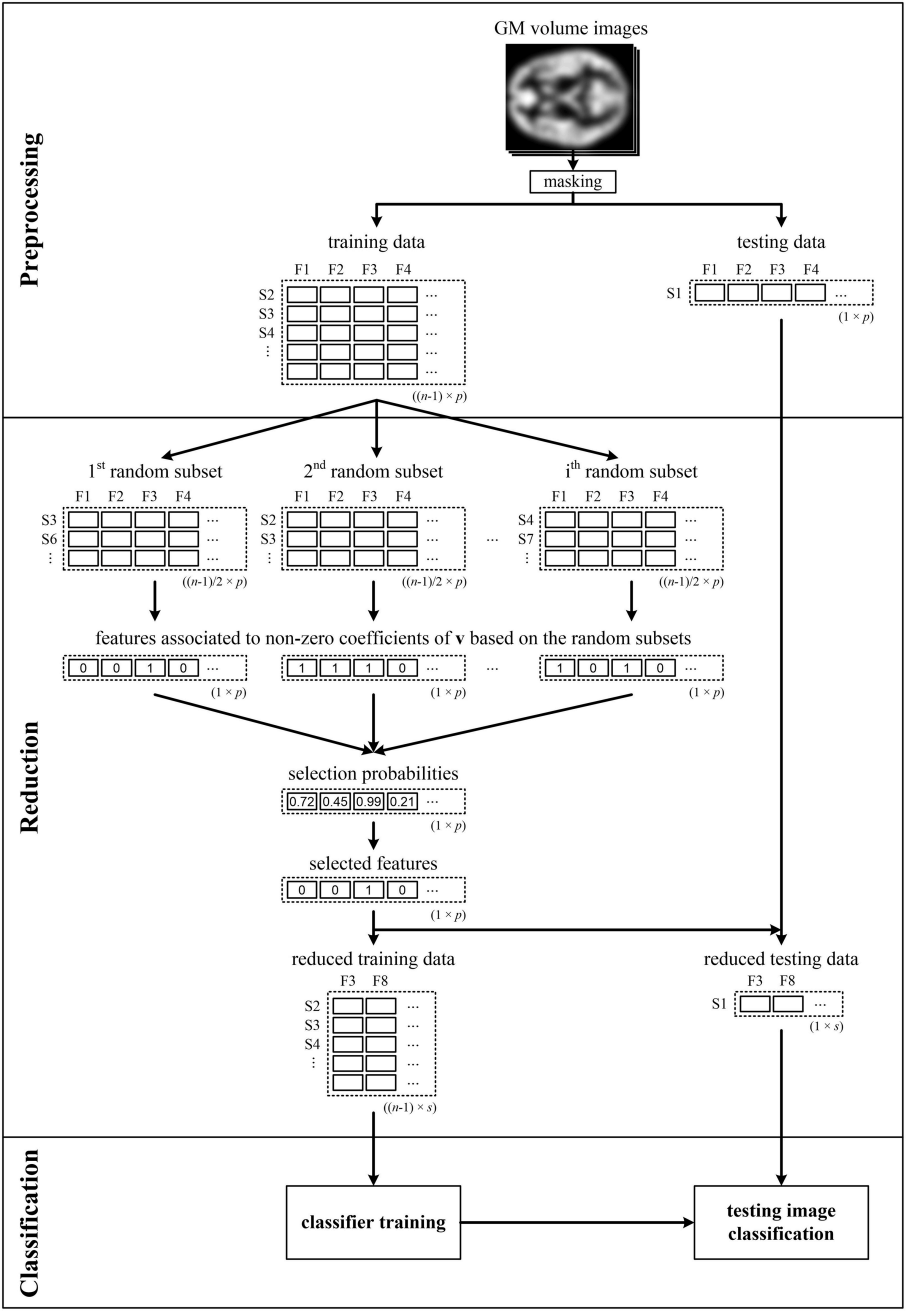
**Illustrative scheme of recognition of patients from controls comprising LOOCV during data reduction and classification—LOOCV^**(rc)**^**. Brain images (e.g., GM volume images), from which the background and non-brain areas are removed using a binary mask, are split into training and testing sets. The training data are used to calculate the selection probabilities of features using pLDA based on random subsets. The training and testing data are then reduced by selecting only the features with selection probabilities ≥0.99. Next, the reduced training data are used to train the classifier, which makes it possible to classify the testing data into a group of schizophrenia patients or HC. The entire process is repeated *n* times as each image is sequentially chosen as the testing image.

It is well-known that data classification as well as data reduction should not use all of the input data simultaneously to avoid overoptimistic classification performance (Guyon and Elisseeff, 2003). Since resampling is performed during data reduction here, and therefore the brain imaging features are selected based on random data subsets, it appears sufficient to perform LOOCV only for feature classification [denoted by LOOCV^(c)^]. To verify this hypothesis, we also performed analysis when we repeatedly split the data into training and testing sets prior to data reduction [marked as LOOCV^(rc)^ and illustrated in Figure 1]. In that case, only the data of *n* − 1 subjects were input into the pLDA with resampling in each LOOCV^(rc)^ iteration, and the data of the testing subject were only used during classification.

All algorithms described in the Materials and Methods Section were implemented in “Penalised Reduction & Classification Toolbox” for MATLAB (the toolbox and documentation are freely available at http://www.iba.muni.cz/index-en.php?pg=research--data-analysis-tools--plda).

## Results

The classification results based on local deformations and GM volumes reduced using pLDA with resampling based on 100 random data subsets and various values of λ, PCA, *t*-test with three thresholds, and the imaging data without reduction are given in Tables 1, 2. In the case of local deformations and GM volumes, the parameter λ ranged from 0.6 to 2.0 and from 0.2 to 1.2, respectively. The pLDA algorithm did not converge for λ > 2 and λ > 1.2, respectively; whereas for λ < 0.6 and λ < 0.2, respectively, it did not reduce the data to less than hundreds of thousands of features. In the case of *t*-test, three thresholds *p* < 0.01, *p* < 0.005, and *p* < 0.001 were used. When we applied a false discovery rate correction, no local deformations remained statistically significant, and only 2000 GM volumes remained significant. On the other hand, a threshold of *p* < 0.05 resulted in too many features (more than 200,000 local deformations and 110,000 GM volumes).

**Table 1:** **Results of LDA and linear SVM (linSVM) classification experiments based on local deformations reduced by pLDA with resampling using various values of λ, PCA, ***t***-test with three thresholds or unreduced**.

**Reduction**	**λ**	**Classifier**	**LOOCV during classification—LOOCV^(c)^**					**LOOCV during reduction and classification—LOOCV^(rc)^**				
			**# features**	**# iterations (mean±SD)**	**Acc (%)**	**Sens (%)**	**Spec (%)**	**# features (mean±SD)**	**# iterations (mean±SD)**	**Acc (%)**	**Sens (%)**	**Spec (%)**
pLDA	0.6	LDA	98,320	3.0 ± 0.0	84.6	86.5	82.7	104,184.2 ± 8527.3	3.0 ± 0.0	65.4	57.7	73.1
		linSVM			86.5	88.5	84.6			69.2	67.3	71.2
pLDA	0.7	LDA	83,193	3.0 ± 0.0	83.7	84.6	82.7	89,008.5 ± 7796.1	3.0 ± 0.0	65.4	57.7	73.1
		linSVM			86.5	88.5	84.6			70.2	67.3	73.1
pLDA	0.8	LDA	71,245	3.0 ± 0.1	82.7	82.7	82.7	76,965.1 ± 7170.8	3.0 ± 0.0	65.4	57.7	73.1
		linSVM			86.5	88.5	84.6			70.2	65.4	75.0
pLDA	0.9	LDA	61,398	2.9 ± 0.3	82.7	82.7	82.7	66,372.6 ± 5736.2	3.0 ± 0.2	66.3	59.6	73.1
		linSVM			86.5	88.5	84.6			64.4	63.5	65.4
pLDA	1.0	LDA	52,927	2.9 ± 0.3	81.7	80.8	82.7	58,355.1 ± 5250.0	2.9 ± 0.3	67.3	61.5	73.1
		linSVM			85.6	86.5	84.6			68.3	65.4	71.2
pLDA	1.1	LDA	45,557	3.1 ± 0.5	82.7	80.8	84.6	50,510.9 ± 4896.3	3.0 ± 0.4	66.3	59.6	73.1
		linSVM			84.6	82.7	86.5			63.5	61.5	65.4
pLDA	1.2	LDA	39,008	3.2 ± 0.6	83.7	82.7	84.6	44,624.4 ± 5221.4	3.0 ± 0.6	66.3	57.7	75.0
		linSVM			84.6	82.7	86.5			66.3	65.4	67.3
pLDA	1.3	LDA	33,082	3.6 ± 0.7	84.6	82.7	86.5	38,712.8 ± 4816.2	3.3 ± 0.6	65.4	55.8	75.0
		linSVM			84.6	82.7	86.5			67.3	65.4	69.2
pLDA	1.4	LDA	27,807	3.9 ± 0.8	84.6	82.7	86.5	33,401.8 ± 4425.2	3.7 ± 0.6	65.4	57.7	73.1
		linSVM			82.7	80.8	84.6			66.3	65.4	67.3
pLDA	1.5	LDA	23,116	4.2 ± 0.8	85.6	82.7	88.5	28,275.0 ± 3403.0	4.1 ± 0.7	68.3	63.5	73.1
		linSVM			81.7	80.8	82.7			63.5	61.5	65.4
pLDA	1.6	LDA	18,917	4.7 ± 0.9	85.6	82.7	88.5	24,092.3 ± 3688.9	4.4 ± 0.7	67.3	61.5	73.1
		linSVM			81.7	80.8	82.7			68.3	67.3	69.2
pLDA	1.7	LDA	15,203	5.0 ± 1.1	84.6	80.8	88.5	19,575.5 ± 3001.0	4.6 ± 0.9	67.3	61.5	73.1
		linSVM			81.7	80.8	82.7			63.5	63.5	63.5
pLDA	1.8	LDA	11,736	5.6 ± 1.5	85.6	80.8	90.4	16,241.5 ± 2996.4	5.0 ± 1.0	63.5	59.6	67.3
		linSVM			80.8	78.8	82.7			63.5	65.4	61.5
pLDA	1.9	LDA	9555	7.1 ± 9.5	83.7	78.8	88.5	12,483.7 ± 2421.6	5.5 ± 1.2	65.4	61.5	69.2
		linSVM			80.8	80.8	80.8			55.8	57.7	53.8
pLDA	2.0	LDA	7306	8.7 ± 13.2	83.7	78.8	88.5	9,910.8 ± 2196.8	6.2 ± 1.4	62.5	59.6	65.4
		linSVM			80.8	78.8	82.7			59.6	59.6	59.6
PCA	–	LDA	–	–	60.6	55.8	65.4	–	–	66.3	65.4	67.3
		linSVM			67.3	61.5	73.1			67.3	65.4	69.2
*t*-test, *p* < 0.01	–	LDA	62,644	–	82.7	82.7	82.7	62,110.1 ± 3241.1	–	66.3	59.6	73.1
		linSVM			84.6	84.6	84.6			70.2	69.2	71.2
*t*-test, *p* < 0.005	–	LDA	38,630	–	84.6	82.7	86.5	38,184.7 ± 2389.9	–	65.4	57.7	73.1
		linSVM			84.6	82.7	86.5			66.3	65.4	67.3
*t*-test, *p* < 0.001	–	LDA	10,860	–	83.7	78.8	88.5	10,671.2 ± 1110.8	–	60.6	57.7	63.5
		linSVM			78.8	76.9	80.8			59.6	63.5	55.8
no	–	LDA	1,924,670	–	67.3	63.5	71.2	–	–	–	–	–
		linSVM			64.4	63.5	65.4			–	–	–

*In columns: the number of selected features, the number of iterations of the pLDA algorithm and cross-validated classification performance measures in percentage—accuracy (Acc), sensitivity (Sens), and specificity (Spec)*.

**Table 2:** **Results of LDA and linear SVM (linSVM) classification experiments based on GM volumes reduced by pLDA with resampling using various values of λ, PCA, ***t***-test with three thresholds or unreduced**.

**Reduction**	**λ**	**Classifier**	**LOOCV during classification—LOOCV^(c)^**					**LOOCV during reduction and classification—LOOCV^(rc)^**				
			**# features**	**# iterations (mean±SD)**	**Acc (%)**	**Sens (%)**	**Spec (%)**	**# features (mean±SD)**	**# iterations (mean±SD)**	**Acc (%)**	**Sens (%)**	**Spec (%)**
pLDA	0.2	LDA	104,565	3.0 ± 0.0	74.0	73.1	75.0	110,082.0 ± 9359.9	3.0 ± 0.0	67.3	65.4	69.2
		linSVM			76.9	76.9	76.9			66.3	63.5	69.2
pLDA	0.3	LDA	75,620	3.1 ± 0.3	74.0	73.1	75.0	80,612.8 ± 7792.0	3.1 ± 0.2	67.3	65.4	69.2
		linSVM			77.9	76.9	78.8			67.3	65.4	69.2
pLDA	0.4	LDA	58,840	3.5 ± 0.5	75.0	73.1	76.9	63,736.4 ± 6748.8	3.3 ± 0.5	67.3	65.4	69.2
		linSVM			77.9	75.0	80.8			66.3	65.4	67.3
pLDA	0.5	LDA	47,535	3.8 ± 0.4	76.0	75.0	76.9	51,974.5 ± 5957.1	3.7 ± 0.5	66.3	65.4	67.3
		linSVM			76.9	73.1	80.8			64.4	63.5	65.4
pLDA	0.6	LDA	38,756	3.9 ± 0.4	76.9	76.9	76.9	42,871.9 ± 5295.7	3.8 ± 0.4	66.3	65.4	67.3
		linSVM			77.9	76.9	78.8			64.4	61.5	67.3
pLDA	0.7	LDA	31,534	4.1 ± 0.4	76.9	76.9	76.9	35,341.3 ± 4742.8	4.0 ± 0.4	66.3	65.4	67.3
		linSVM			78.8	76.9	80.8			63.5	59.6	67.3
pLDA	0.8	LDA	25,253	4.3 ± 0.5	76.9	76.9	76.9	28,857.4 ± 4245.3	4.2 ± 0.4	66.3	65.4	67.3
		linSVM			78.8	76.9	80.8			63.5	57.7	69.2
pLDA	0.9	LDA	19,670	4.6 ± 0.6	76.9	76.9	76.9	22,762.5 ± 4087.9	4.4 ± 0.6	68.3	67.3	69.2
		linSVM			80.8	80.8	80.8			64.4	63.5	65.4
pLDA	1.0	LDA	14,923	4.7 ± 0.6	77.9	78.8	76.9	18,001.1 ± 3351.3	4.6 ± 0.6	67.3	65.4	69.2
		linSVM			79.8	80.8	78.8			59.6	55.8	63.5
pLDA	1.1	LDA	10,596	4.9 ± 0.6	80.8	80.8	80.8	13,417.8 ± 2828.5	4.8 ± 0.7	65.4	63.5	67.3
		linSVM			75.0	73.1	76.9			58.7	55.8	61.5
pLDA	1.2	LDA	6543	5.4 ± 1.7	83.7	84.6	82.7	9,624.9 ± 2371.7	5.0 ± 0.9	65.4	61.5	69.2
		linSVM			77.9	75.0	80.8			59.6	53.8	65.4
PCA	–	LDA	–	–	57.7	55.8	59.6	–	–	59.6	57.7	61.5
		linSVM			69.2	69.2	69.2			59.6	57.7	61.5
*t*-test, *p* < 0.01	–	LDA	44,168	–	76.0	75.0	76.9	43,716.8 ± 3122.2	–	67.3	67.3	67.3
		linSVM			76.9	73.1	80.8			65.4	65.4	65.4
*t*-test, *p* < 0.005	–	LDA	28,638	–	76.9	76.9	76.9	28,252.1 ± 2331.2	–	67.3	67.3	67.3
		linSVM			79.8	78.8	80.8			69.2	65.4	73.1
*t*-test, *p* < 0.001	–	LDA	9470	–	79.8	80.8	78.8	9,316.1 ± 1134.6	–	67.3	63.5	71.2
		linSVM			72.1	71.2	73.1			58.7	57.7	59.6
no	–	LDA	748,931	–	63.5	63.5	63.5	–	–	–	–	–
		linSVM			64.4	61.5	67.3			–	–	–

*In columns: the number of selected features, the number of iterations of the pLDA algorithm and cross-validated classification performance measures in percentage—accuracy (Acc), sensitivity (Sens), and specificity (Spec)*.

**Figure 2:**
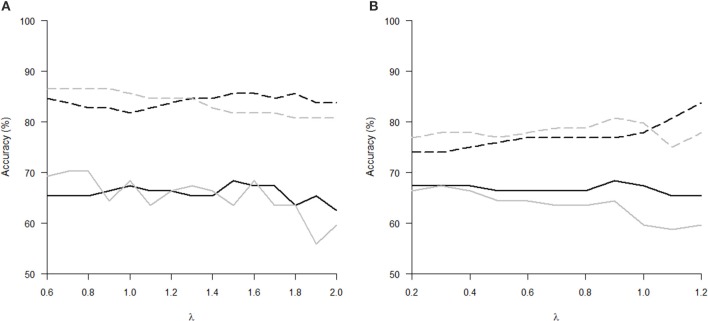
**Classification accuracy versus the parameter λ of pLDA with resampling in experiments with two types of imaging features: (A) local deformations, (B) GM volumes**. The black solid line represents LDA classifier with LOOCV^(rc)^, the gray solid line stands for linear SVM classifier with LOOCV^(rc)^, the black dashed line depicts LDA with LOOCV^(c)^, and the gray dashed line denotes linear SVM with LOOCV^(c)^.

The Tables 1, 2 show that increasing λ led to a decreased number of selected discriminating features and an increased number of iterations of the minorization-maximization algorithm within pLDA. The classification performance differed considerably according to type of LOOCV that we performed. The median accuracy of the classification of local deformations using LDA was 83.7% (range: 81.7–85.6%) in the case of LOOCV^(c)^ compared with 65.4% (range: 62.5–68.3%) in the case of LOOCV^(rc)^. The linear SVM classification of local deformations led to similar median accuracies of 84.6% (range: 80.8–86.5%) and 66.3% (range: 55.8–70.2%), respectively. The classification of GM volumes based on LOOCV^(c)^ led to slightly less overestimated results compared with local deformations; the median accuracy of classification was 76.9% (range: 74.0–83.7%) and 77.9% (range: 75.0–80.8%) using LDA and linear SVM, respectively, compared with LOOCV^(rc)^ with median accuracies of 66.3% (range: 65.4–68.3%) and 64.4% (range: 58.7–67.3%), respectively. For better illustration, classification accuracy achieved in the experiments vs. the values of the parameter λ was visualized (Figure 2).

**Figure 3:**
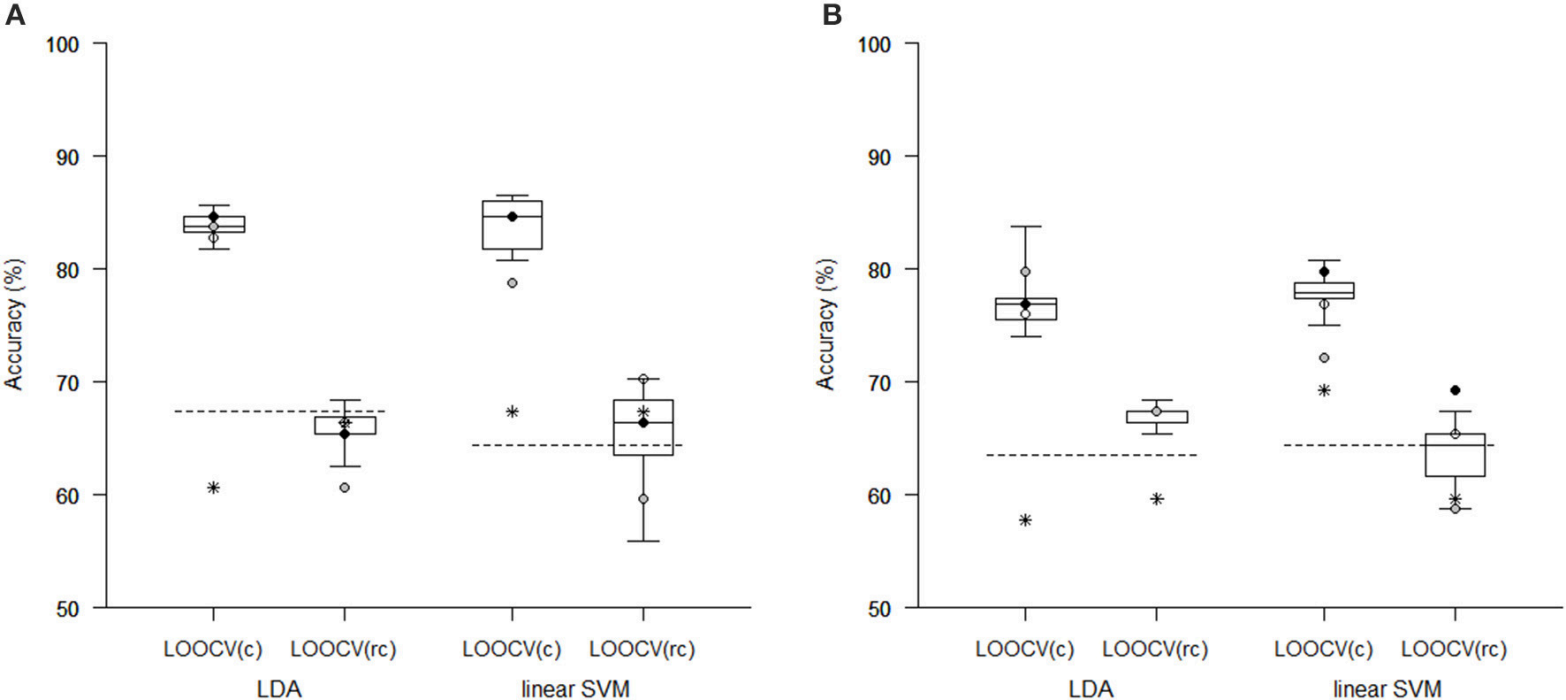
**Comparison of classification accuracy achieved in experiments with two types of imaging features: (A) local deformations, (B) GM volumes**. The features were reduced using pLDA with resampling (the boxplots show the range of accuracy for different values of λ), using PCA (stars), and using a *t*-test with a threshold of either *p* < 0.01 (empty circles), *p* < 0.005 (filled black circles), or *p* < 0.001 (filled gray circles). After reduction, the features were then classified using LDA or linear SVM while performing LOOCV during data classification [LOOCV^(c)^] or during data reduction and classification [LOOCV^(rc)^]. Classification accuracies obtained with original unreduced data are represented by dashed lines.

Comparison of the reduction methods showed that the maximum accuracy achieved in the classification of data reduced using pLDA with resampling was higher than the accuracy of the classification of data reduced by PCA or a *t*-test in six out of eight of the classification experiments (Tables 1, 2, Figure 3). However, the difference in accuracy was quite small and statistically insignificant (based on McNemar's test) apart from the cases of reduction using PCA in the case of LOOCV^(c)^, classification of GM volumes reduced by a *t*-test with *p* < 0.01 and *p* < 0.005 using LDA with LOOCV^(c)^; and *t*-test with *p* < 0.001 for the second, third, fourth, and seventh experiment (the order corresponds to the Figure 3) showing that too strict threshold of *t*-test leading to low number of selected voxels results in low classification performance. In the case of linear SVM with LOOCV^(rc)^, the classification of data reduced by pLDA yielded a lower accuracy than the classification of GM volumes reduced with a *t*-test with *p* < 0.005 and same accuracy as the classification of local deformations reduced with *t*-test with *p* < 0.01. When comparison with unreduced data was performed, the maximum accuracy achieved in the classification of data reduced using pLDA with resampling was higher than the accuracy obtained in the classification of unreduced data in all eight experiments. However, the differences were statistically significant only in the case of LOOCV^(c)^. Finally, comparison of the achieved classification accuracies with classification by chance was also performed (using one-sample binomial test), showing that classification results obtained by PCA, *t*-test and maximum classification performance achieved by pLDA was statistically significantly better than chance in all experiments except for PCA on GM volumes with LOOCV^(c)^ and *t*-test with *p* < 0.001 on GM volumes with LOOCV^(rc)^.

**Figure 4:**
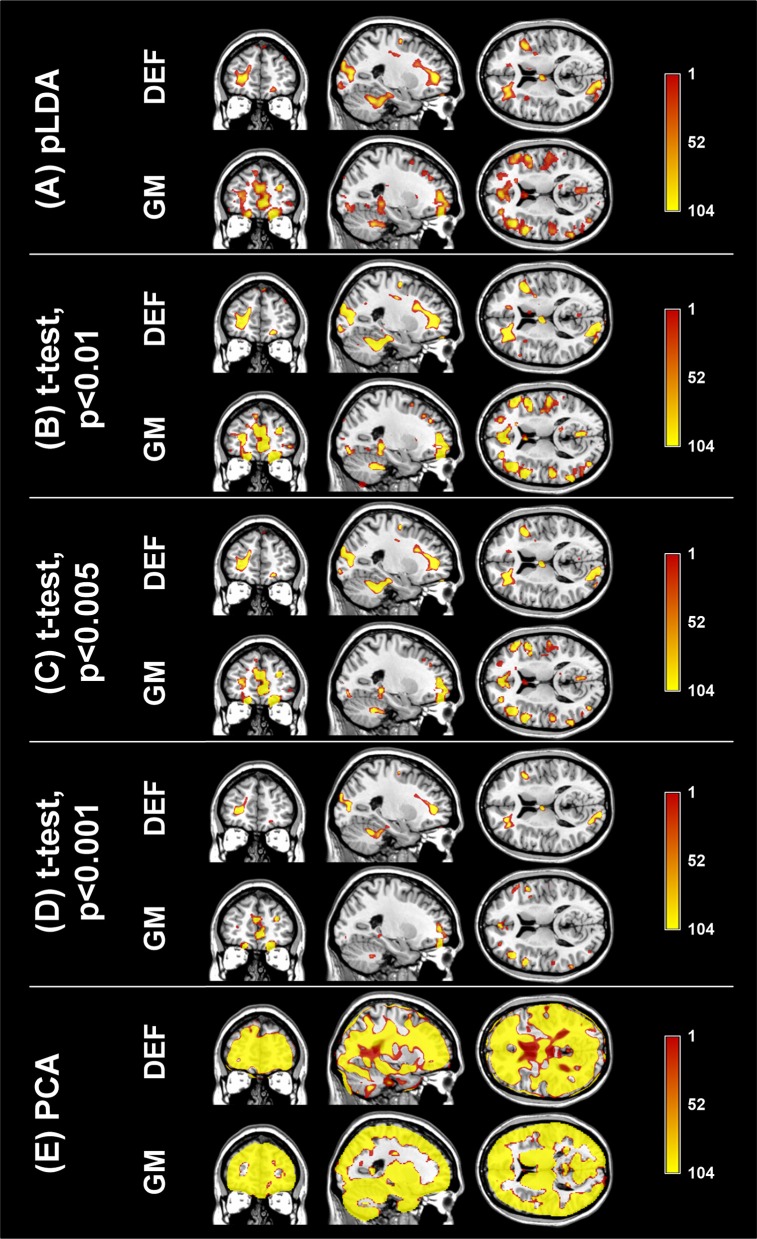
**Coronal, sagittal, and transversal slices showing the automatically detected strongly discriminative features in local deformations (DEF) and gray matter volumes (GM) using: (A) pLDA with resampling (for λ = 1.6 and λ = 0.9, respectively), (B) ***t***-test with ***p*** < 0.01, (C) ***t***-test with ***p*** < 0.005, (D) ***t***-test with ***p*** < 0.001, (E) PCA**. The color coding corresponds to how many times each feature was selected as one of the discriminative features in the case of pLDA with resampling and *t*-test or contributed most to the first principal component in the case of PCA within all 104 LOOCV^(rc)^ iterations.

**Figure 5:**
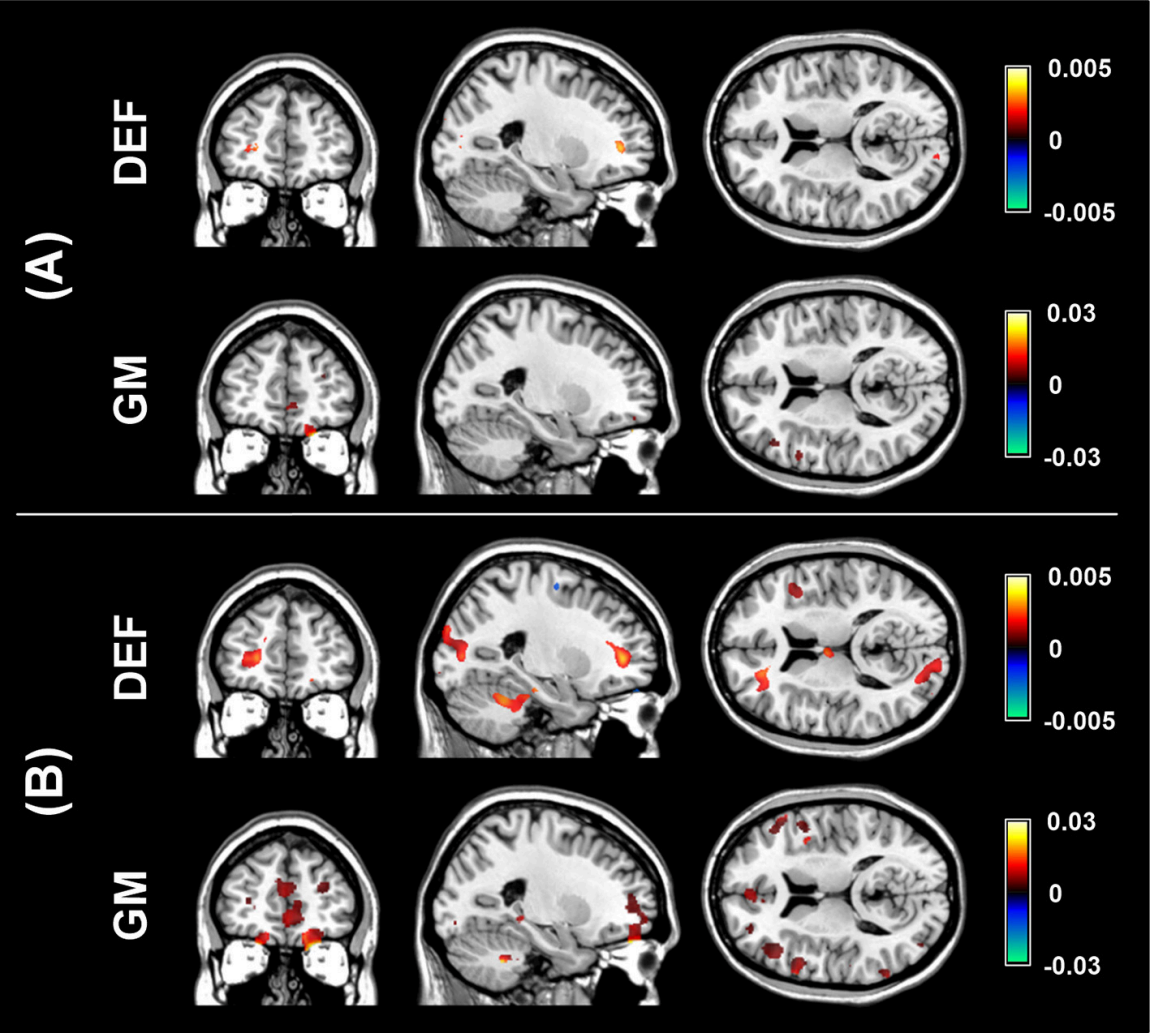
**Visualization of mean discriminative weights of local deformation (DEF) and gray matter (GM) volume features selected by pLDA with resampling (for λ = 1.6 and λ = 0.9, respectively) in: (A) all LOOCV^**(*rc*)**^ iterations, (B) more than half of LOOCV^**(*rc*)**^ iterations**. The mean weights were calculated as an average of non-zero coefficients of **v** over 100 resampling iterations and 104 LOOCV^(rc)^ folds.

The aim of the pLDA algorithm is to identify the features that best discriminate between schizophrenia patients and HC. Figure 4 shows a visualization of the selected strongly discriminating local deformations and GM volume features. It must be noted that in the case of LOOCV^(rc)^, slightly different sets of selected features were obtained in each LOOCV iteration. Thus, the color coding in Figure 4 corresponds to the number of LOOCV iterations in which the feature was among the most discriminative features. In the case of local deformations, the discriminative features form connected regions in the left prefrontal cortex, the right anterior insula, the medial parts of the thalamus, and the cerebellar cortex. The discriminative GM volume features lie also in these brain regions as well as in the inferior frontal gyrus, the anterior cingulum, the hippocampus, and parahippocampal gyrus, the caudate nucleus, and the superior and middle temporal gyrus. Similar brain areas were identified as the most discriminative ones also using *t*-test. In the case of PCA, the features whose loadings contributed most to the first principal component (based on a threshold at 30% of maximum absolute value of the loadings) covered almost entire brain or gray matter areas, respectively. To further investigate discriminative capability of features selected by pLDA with resampling in all as well as in more than half of LOOCV^(rc)^ folds, mean discriminative weights (specifically, mean of non-zero weights over 100 resampling iterations and 104 LOOCV^(rc)^ folds) were calculated and visualized (Figure 5).

## Discussion

In our experiment with T1-weighted MRI data of 52 FES patients and 52 HC, we found that pLDA with resampling, which is a supervised, multivariate, whole-brain reduction technique, did not yield a significantly better classification performance than unsupervised, multivariate reduction using PCA or mass univariate selection using a *t*-test as well as unreduced data in the case of LOOCV^(rc)^. If LOOCV was performed only during classification, pLDA yielded significantly better classification accuracy than PCA because the pLDA results were considerably optimistically biased even though we performed resampling within the pLDA algorithm. Our results also showed that PCA was less prone to overtraining in the case of LOOCV^(c)^ because PCA does not use labels during data reduction compared with pLDA and a *t*-test. So, the results indicate that LOOCV^(c)^ is incorrect and lead to biased classification results (especially in the case of supervised data reduction methods despite of performed resampling within the reduction methods) and LOOCV^(rc)^ must be used instead.

In our experiment, we also investigated how data preprocessing influenced our classification results. We used two types of features obtained by different preprocessing of the MRI data, namely local deformations and GM volumes. We decided not to use the MRI intensities since they yielded considerably poorer classification results compared with the two other types of features in our previous study (Janousova et al., 2015). Here, we achieved similar performances using LOOCV^(rc)^ classification based on local deformations and GM volumes reduced using pLDA. The same was true for data reduced using a *t*-test. In the case of reduction using PCA, local deformations enabled a more accurate classification than was possible using GM volumes. When we performed LOOCV^(c)^, the GM volumes reduced by pLDA as well as a *t*-test led to slightly less overestimated results than in local deformations. The most discriminating local deformation features that were automatically detected by pLDA with resampling were located in the left prefrontal cortex, the cerebellar cortex, the medial parts of the thalamus and the right anterior insula. Furthermore, the most discriminating GM volume features also formed clusters in the inferior frontal gyrus, the anterior cingulum, the hippocampus and parahippocampal gyrus, and the superior and middle temporal gyrus. All of these results are consistent with the findings published in previous studies (Wright et al., 2000; Shenton et al., 2001; Niznikiewicz et al., 2003; Honea et al., 2005; Ellison-Wright et al., 2008). Moreover, it is known that these brain areas are involved in higher cognitive, integrative and regulatory functions that are impaired in schizophrenia (Niznikiewicz et al., 2003; Antonova et al., 2004). For example, prefrontal anomalies have been involved in negative symptoms and cognitive impairments such as deficits in working memory and executive functions (Goldman-Rakic and Selemon, 1997). The validity of the selected most discriminative brain regions was also confirmed by *t*-test. Unfortunately, a direct comparison of areas of most discriminating local deformations and GM volumes is not possible because of different resolution of the two types of images. Nevertheless, the comparison of brain areas was not a goal of this study. We aimed at using imaging data at their highest possible resolution and we wanted to avoid loss of information in the local deformations by resampling the deformation images to 1.5 × 1.5 × 1.5 mm. Regarding the features whose loadings contributed most to the first principal component, they covered almost entire brain or gray matter areas. Even if the thresholding of the loadings was stricter leading to smaller brain regions, those regions could not be considered as the discriminative ones because PCA does not aim for identification of discriminative features but rather for efficient data representation while preserving maximum amount of variance without regard to data labels (Kasparek et al., 2011, Janousova et al., 2015).

We additionally examined classification performance using two most common classification methods: LDA and linear SVM. We used LDA here for classification since we wished to ensure that our results would be comparable with other studies despite the fact that results obtained using LDA might be unstable in cases with a limited number of subjects for analysis (Thomaz et al., 2007). We also used linear SVM because of comparability with other studies and because we wished to avoid overtraining, which can arise when nonlinear kernels are used (Mourao-Miranda et al., 2012). Performing only the experiments with LOOCV during classification because a reduction using pLDA contained resampling that should prevent overestimating the results, we could state that LDA and linear SVM yield similar classification results (maximum accuracies of 85.6% (82.7% sens., 88.5% spec.) and 86.5% (88.5% sens., 84.6% spec.), respectively, for the classification of local deformations; and maximum accuracies of 83.7% (84.6% sens., 82.7% spec.) and 80.8% (80.8% sens., 80.8% spec.), respectively, for the classification of GM volumes). Such classification performance is superior to previous studies based on LDA that reported classification accuracies ranging from 70.7 to 82.9% based on MRI data (Leonard et al., 1999; Nakamura et al., 2004; Takayanagi et al., 2010; Karageorgiou et al., 2011; Ota et al., 2012) as well as a meta-analytical sensitivity of 76.4% (95% CI: 71.9–80.4%) and specificity of 79.0% (95% CI: 74.6–82.8%) based on 20 structural MRI studies (Kambeitz et al., 2015). Nevertheless, such LOOCV^(c)^ approach is incorrect. Thus, we also performed experiments with LOOCV used during data reduction and classification and we achieved poorer results; for the classification of local deformations using LDA and linear SVM, the maximum accuracy was 68.3% (63.5% sens., 73.1% spec.) and 70.2% (67.3% sens., 73.1% spec.), respectively. For the classification of GM volumes, the maximum accuracy that we achieved was 68.3% (67.3% sens., 69.2% spec.) and 67.3% (65.4% sens., 69.2% spec.), respectively. Classification based on data reduced using PCA or a *t*-test was even slightly less accurate apart from the classification of GM volumes reduced with a *t*-test with *p* < 0.005 using linear SVM with LOOCV^(rc)^ with an accuracy of 69.2% (65.4% sens., 73.1% spec.).

The reason why our classification performance with LOOCV performed during data reduction and classification is so limited may be due to the fact that schizophrenia is not a single disease entity but instead a group of phenotypically similar disorders (although the current knowledge is not sufficient to isolate these disorders; Silveira et al., 2012). Another possibility is that our cohort of patients contained subjects with a future episodic course who could not be distinguished from healthy individuals (Mourao-Miranda et al., 2012). Such heterogeneity in data samples causes an overlap in discriminative features between patients and controls which leads to a drop of classification accuracy (Schnack and Kahn, 2016). Another reason explaining the limited classification performance of our results might be that the findings of other studies were biased because authors used a mixture of first-episode and chronic schizophrenia patients, a combination of male and female subjects, a small number of subjects, and/or incorrect classification. It is already known that the statistical power of neuroscience studies is often very low and that the effect size is frequently overestimated (Button et al., 2013). To avoid optimistically biased results, we used only patients with a first episode of schizophrenia in our study; medication affects whole-brain GM volume (Ho et al., 2011), and therefore brain anatomy abnormalities observed at the first episode of schizophrenia are likely to be much more subtle relative to chronic schizophrenia (Ellison-Wright et al., 2008). Therefore, the classification performance can be overestimated if the data set contains chronic schizophrenia patients in addition to FES patients. For example, a previous study recovered an accuracy of 90.8% for male subjects and 91.8% for female subjects while discriminating a mixed group of FES and chronic patients from healthy individuals using the COMPARE algorithm (Fan et al., 2007); whereas the same algorithm yielded an accuracy of 73.4% for the classification of only FES and healthy subjects (Zanetti et al., 2013). Furthermore, to reduce bias in our results, we used only males in our study because it has been shown that there are differences in structural brain abnormalities in males and females (Nasrallah et al., 1990). In terms of the data sets used in classification experiments, an independent validation set should be used ideally (Nieuwenhuis et al., 2012; Schnack and Kahn, 2016). When no external validation set is available (which is unfortunately frequent in neuroimaging studies), Nieuwenhuis et al. (2012) recommend using more than 130 subjects as small number of subjects can lead to unstable classification models. Even though our study does not include such a large number of subjects, our data set containing 104 subjects is still much larger than the data sets used in many previously published MRI-based classification studies of schizophrenia (Leonard et al., 1999; Kawasaki et al., 2007; Sun et al., 2009; Takayanagi et al., 2010; Karageorgiou et al., 2011; Kasparek et al., 2011; Borgwardt et al., 2012; Pettersson-Yeo et al., 2013). Concerning the influence of type of LOOCV, apart from our current study in which results based on LOOCV performed only during classification are noticeably overestimated, another example is in study of Nieuwenhuis et al. (2012) that achieved an accuracy of 86.8% in their discovery sample while selecting the top 10% of features ranked based on the absolute values of the elements of the weight of the SVM vector compared with an accuracy of 69.1% obtained using an independent validation data set based on the selected features. When no data reduction was performed, these authors obtained similar accuracies in their discovery sample (71.4%) and validation sample (70.4%).

Our current results, as well as a meta-analysis by Kambeitz et al. (2015), suggest that even though much work has focused on using neuroimaging data to distinguish schizophrenia patients from healthy individuals, the classification performance is still too low to be considered for clinical application. Therefore, researchers should focus on continuing schizophrenia research and improving as well as inventing data reduction and classification methods to attain better classification performances; early and accurate diagnoses have the ability to shorten the duration of untreated psychosis and therefore improve therapeutic outcomes and overall patient prognoses (Perkins et al., 2005). One way of improving classification performance may be to use different imaging modalities, such as diffusion tensor imaging (DTI) or functional MRI. These modalities are capable of revealing information about connections inside the brain which could be of help in classification as schizophrenia is characterized by a deficit of interconnections (Michael et al., 2008). A promising accuracy of 96% for the classification of DTI data has already been reported (Ardekani et al., 2011), but the results may be a bit too optimistic since the authors used a mixture of FES and chronic patients and numbers of patients and controls were low (25 subjects in each group). Resting-state functional MRI also exhibits promising results since its sensitivity was higher than that of structural MRI studies (Kambeitz et al., 2015). However, the resting-state functional MRI studies were based on quite small samples; while using large samples, the classification accuracy will probably decrease (Schnack and Kahn, 2016). To conclude, even though the classification performance of schizophrenia classification studies is still too low for application in schizophrenia diagnostics, schizophrenia research should continue with future studies performed on large samples free of chronic schizophrenia patients using correctly designed and performed classification procedures.

## Author contributions

EJ designed the study, performed the analysis, and wrote the manuscript. GM participated in the reduction algorithm design and the manuscript writing. TK contributed to study design, collected the data and participated in interpretation of analysis results. DS contributed to study design, performed image data preprocessing and critically revised the manuscript. All authors read and approved the final manuscript.

### Conflict of interest statement

The authors declare that the research was conducted in the absence of any commercial or financial relationships that could be construed as a potential conflict of interest.
